# Efficient Prediction of Progesterone Receptor Interactome Using a Support Vector Machine Model

**DOI:** 10.3390/ijms16034774

**Published:** 2015-03-03

**Authors:** Ji-Long Liu, Ying Peng, Yong-Sheng Fu

**Affiliations:** College of Veterinary Medicine, South China Agricultural University, Guangzhou 510642, China; E-Mails: yingpeng2013@126.com (Y.P.); yshf2013@126.com (Y.-S.F.)

**Keywords:** protein–protein interaction, support vector machine, progesterone receptor

## Abstract

Protein-protein interaction (PPI) is essential for almost all cellular processes and identification of PPI is a crucial task for biomedical researchers. So far, most computational studies of PPI are intended for pair-wise prediction. Theoretically, predicting protein partners for a single protein is likely a simpler problem. Given enough data for a particular protein, the results can be more accurate than general PPI predictors. In the present study, we assessed the potential of using the support vector machine (SVM) model with selected features centered on a particular protein for PPI prediction. As a proof-of-concept study, we applied this method to identify the interactome of progesterone receptor (PR), a protein which is essential for coordinating female reproduction in mammals by mediating the actions of ovarian progesterone. We achieved an accuracy of 91.9%, sensitivity of 92.8% and specificity of 91.2%. Our method is generally applicable to any other proteins and therefore may be of help in guiding biomedical experiments.

## 1. Introduction

Proteins determine the phenotype of all organisms. Typically, proteins are not functional in isolated forms; it has been estimated that over 80% of all proteins do not operate alone but in complexes through protein–protein interaction (PPI) [1]. Uncovering PPI helps to elucidate protein functions and further understand various biological processes in the cell. PPI is also a critical regulatory event in pathology [2]. Inappropriate PPI is linked to many diseases and therefore represents an important target for drug discovery [3].

Various experimental techniques have been developed for large scale PPI analysis, such as yeast-two-hybrid (Y2H) and affinity purification combined with mass spectrometry (APMS). The limitation of these approaches is that they have experienced high rates of noise and false positives [4]. In addition, these approaches are time-consuming and expensive. As a result, experimentally-determined PPI pairs currently cover only a small fraction of the complete PPI space [5,6]. Although recent technical improvements are expected to increase the confidence of results and lower the costs, identification of PPI with high coverage and quality remains a challenge. Alternatively, computational methods have been proposed for PPI prediction. In general, these methods fall into two categories, those based on structural information of proteins, and those based on primary sequences [7]. Several groups have demonstrated the feasibility of these methods in the prediction of genome-wide PPI network in model organisms, such as yeast [8,9] and human [9,10,11,12].

So far, most computational studies are intended for pair-wise prediction of PPI in the whole genome. Theoretically, predicting protein partners for a single protein is likely a simpler problem. Given enough data for a particular protein, the results can be more accurate than general PPI predictors. In the present study, we developed a machine learning method based on a support vector machine (SVM) with selected features centered on a particular protein. As a proof-of-concept study, we applied this method to predict the interactome of progesterone receptor (PR), a protein which is essential for coordinating female reproduction in mammals by mediating the actions of the ovarian progesterone [13]. Our method was more accurate than general PPI models. It will be useful in guiding biomedical experiments.

## 2. Results

### 2.1. Performance Evaluation of the Support Vector Machine (SVM) Model

The goal of the present study was to assess the potential of efficient prediction of PPI partners for a particular protein using a machine learning approach. Our method was developed using a support vector machine (SVM), which has been widely adopted in predicting PPI [7]. Given a data set in which each sample is characterized by an *n*-dimensional vector, an SVM learns a boundary between positive and negative samples with maximum margin. The remaining uncharacterized samples in the data set are then classified according to the decision boundary.

As a proof-of-principle study, the progesterone receptor (PR) protein was chosen. PR is a ligand-activated transcription factor. It interacts with many associated factors, forming a typical protein complex [13]. To construct the positive training set, we searched the PINA2 database and identified 69 non-redundant PR-interacting proteins. A negative training set of the same size was randomly chosen from the whole genome. Random selection was employed because we expected a sizable proportion of proteins in the whole genome were not physiologically relevant to PR. The final training data set included 69 PR-interacting protein and 68 non-interacting proteins (Table S1).

For predicting PPI by sequences, one of the main computational challenges is to find a suitable way to fully describe the important information of PPI. To solve this problem, we proposed a descriptor which considered both amino acid composition and functional domain annotation. Finally, a 433-dimension feature vector (20 for amino acids plus 413 for functional domains) was built to represent each protein. We employed an SVM to generate computational models incorporating these features. The radial basis function (RBF) was selected as the kernel function. To validate the prediction performance in a self-consistent way, 5-fold cross validation was carried out. We evaluated the performance of the SVM model in terms of sensitivity, specificity and accuracy.

(1) $$Sensitivity=\frac{TP}{TP+FN}$$

(2) $$Sepecificity=\frac{TN}{TN+FP}$$

(3) $$Accuracy=\frac{TP+TN}{TP+FP+TN+FN}$$

True positives (TP) were actual interacting proteins that were predicted correctly. True negatives (TN) were non-interacting proteins that were predicted correctly. False positives (FP) were non-interacting proteins that were predicted as interacting proteins. False negatives (FN) were interacting proteins that were missed.

**Figure 1:**
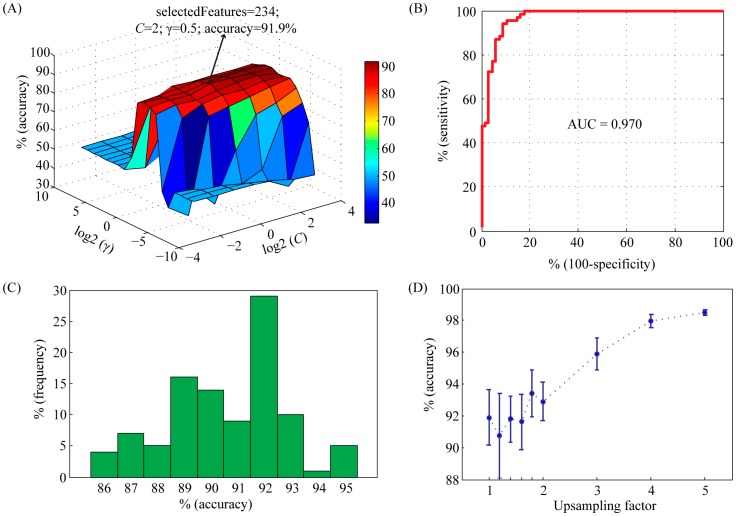
Characteristics of support vector machine (SVM) classifier with radial basis function (RBF) kernel on training set. (**A**) Accuracy surface of 5-fold crossover validation on training set *versus* the variations of parameters *C* and γ. The best accuracy was achieved at *C* = 2 and γ = 0.5. The number of selected features was 234; (**B**) The receiver-operating characteristic (ROC) curve and corresponding area under the curve (AUC) statistics for the SVM classifier with the parameters *C* = 2 and γ = 0.5; (**C**) Histogram showing the influence of randomly selected negative data set on the performance of SVM model. The selection procedure was repeated 100 times; (**D**) The importance of upsampling on the performance of SVM model. The positive data set was upsampled by a factor from 1 to 5. Then a balanced negative data set was constructed by random selection. For each upsampling factor, negative data set selection was repeated 10 times. Accuracy values were shown as mean ± std.

In order to achieve good results, the capacity parameter *C* and the kernel parameter γ of the SVM were tried using a grid search method in the range of *C* = 2^−4^, 2^−3^, …, 2^4^ and γ = 2^−10^, 2^−9^, …, 2^10^, respectively. Meanwhile, feature selection was performed during parameter optimization. The accuracy profile of the 5-fold cross validation on the training set *versus* the variations of parameters *C* and γ was shown in Figure 1A. Obviously, the prediction accuracy had a maximum peak at (*C*, γ) = (2, 0.5) with 234 selected features, indicating that the optimal values of *C* and γ for constructing SVM model were 2 and 0.5, respectively. With these parameters, we used the receiver operating characteristic (ROC) curve to present the inter-relationship between specificity and sensitivity (Figure 1B). The area under the curve (AUC) statistics provides a useful metric for the performance of a classifier. Whereas an AUC value close to 1 indicates an excellent classifier, a curve that lies close to the diagonal (AUC = 0.5) has no information content. Our classifier achieved an AUC of 0.970. At the maximum point of accuracy (91.9%), the sensitivity and specificity were 92.8% and 91.2%, respectively.

In order to analyze the influence of negative training set on the performance of SVM model, we repeated the random selection process 100 times. The average accuracy was 91.8% (ranging from 85.8% to 95.4%) (Figure 1C), suggesting that our model was very robust to random selection of negative data set. In order to overcome overfitting, we also generated larger negative training sets. An upsampling factor of 1 to 5 was used. The accuracy was significantly improved when the upsampling factor was larger than 3 (Figure 1D).

**Figure 2:**
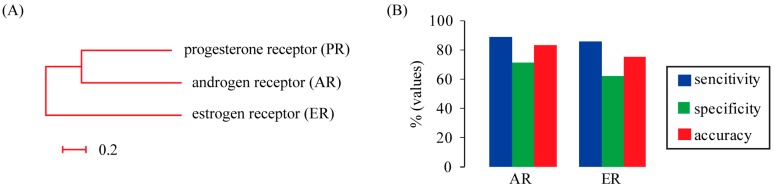
The performance of SVM classifier on different test sets. (**A**) The close homology between progesterone receptor (PR), androgen receptor (AR) and estrogen receptor (ER). The phylogenetic tree was constructed using maximum-likelihood method based on human protein sequences. The scale bar below the tree indicates amino acid change frequency; (**B**) SVM performance on independent data sets of AR and ER.

Androgen receptor (AR) and estrogen receptor (ER), together with PR, are members of steroid receptors [14]. They are considered to share a common ancestor (Figure 2A). In humans, the similarity of PR to AR is 35% and PR to ER is 14%. It has been shown that all steroid receptors recruit a similar set of cofactors [15]. Therefore, we prepared two independent test sets based on AR- and ER-interacting proteins. The performance of our SVM model was summarized in Figure 2B. The prediction accuracy values for AR and ER data sets achieved by our method were 81.5% and 78.2%, respectively. The ER data set had a relatively lower accuracy, which may reflect the relatively lower homology of ER to PR compared with AR. These results demonstrated that our model trained on the PR dataset was also able to predict AR and ER partners with high accuracy.

### 2.2. Complete Scan of the Human Proteome for PR-Binding Proteins

**Figure 3:**
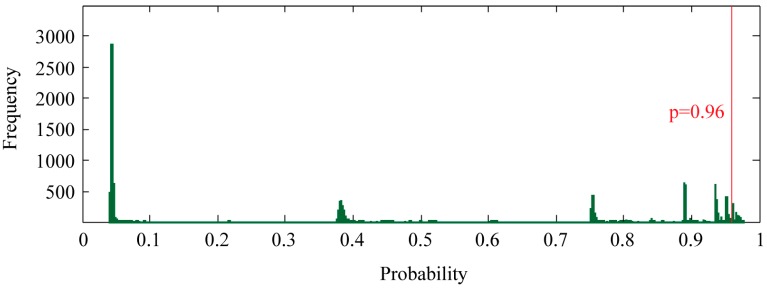
Genome-wide prediction of PR-interacting proteins. SVM classification probability, which measures the confidence of the prediction, was shown as frequency histogram. Bin width of 0.1% was used. PR-interacting proteins were selected using a probability threshold of 0.96.

To find new proteins that potentially interact with PR, we ran our SVM model against all human protein records in the UniProt database. The proteins in the training set were excluded. During prediction, the class probability estimated by the SVM was enabled, providing a convenient measure of prediction confidence. Of all 17,847 proteins, the SVM model predicted 827 non-redundant proteins as potential candidates by a threshold of *p* ≥ 0.96 (Figure 3) (Table S2). This threshold was selected because all correctly classified proteins in the training set exhibited *p* ≥ 0.96.

**Figure 4:**
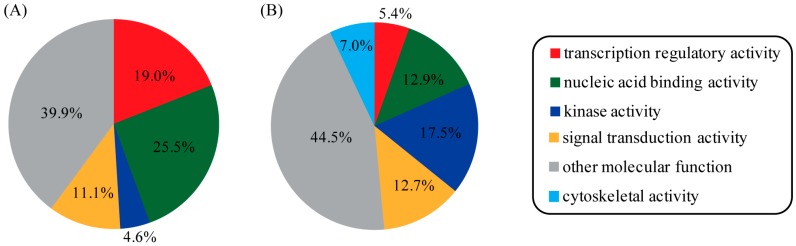
Functional clustering analysis of PR-interacting proteins. Proteins were mapped to GOslim terms under the molecular function category. This analysis was applied on the two different protein lists: (**A**) known PR-interacting proteins and (**B**) newly predicted PR-interacting proteins.

The known and newly predicted PR-interacting proteins were assigned to molecular functional categories according to the annotation from gene ontology. It turned out that known PR-interacting proteins showed a strong preference for transcription regulatory activity, nucleic acid binding activity, kinase activity, and signal transduction activity (Figure 4A). As expected, most of the newly predicted PR-interacting proteins fell into the same functional categories. However, a lower portion of proteins with transcription regulatory activity and a larger portion of proteins with kinase activity were observed in newly predicted PR-interacting proteins compared to known ones. Interestingly, we discovered that a novel category termed cytoskeletal activity was unique to predicted PR-interacting proteins (Figure 4B). This category might provide new clues for PR regulation and function, thus deserving further investigation.

## 3. Discussion

Protein-protein interaction (PPI) is essential for almost all cellular processes and identification of PPI is a crucial task for biomedical researchers. So far, several computational tools have been developed for genome-wide prediction of PPI using general pair-wise features in various model organisms. However, for biologists, they are usually interested in getting a list of potential PPI partners for a single protein. Theoretically, predicting protein partners for a particular protein is likely a simpler problem and the results can be more accurate than general PPI predictors, as protein-specific information can be used. As a proof-of-concept study, we applied this method to predict the interactome of progesterone receptor (PR), a protein which is essential for coordinating female reproduction in mammals by mediating the actions of ovarian progesterone. Previously, Li *et al.* [16] reported a PPI prediction system using pair-wise features with a very high performance. The sensitivity, specificity and accuracy were 89.17%, 92.17% and 90.67%, respectively. However, when applied to our PR dataset, their method achieved an accuracy of 60.14%. Although the sensitivity was 95.71%, the specificity was as low as 23.53% due to a large number of false positives. In the present study, we developed a machine learning method based on an SVM with selected features centered on a particular protein. Our model had a balanced performance: an accuracy of 91.9%, sensitivity of 92.8% and specificity of 91.2%. In order to see the general effectiveness, we applied our method to another 10 proteins, three of which are transcription factors (STAT3, MYC and E2F1). The average accuracy was 89.5% (Figure S1). No preference for transcription factors was observed. These data suggest that our method can be used in a variety of applications.

Our model was built merely on protein sequences. In fact, the majority of existing PPI prediction methods are based on protein sequences but not protein structures [7]. Although structure-based models can provide further details of residual-level PPI interface, they are hindered by the unavailability of complete 3D structure data for most proteins. As in the case of PR, only the ligand binding domain and the DNA binding domain at the *C*-terminal were structurally determined [17,18], whereas the activation function domain at the *N*-terminal which is believed to be the main interface of PPI has not been determined yet. Structure-based models are apparently not suitable for PR. In the present study, we extracted 433 features for each protein (20 for amino acids plus 413 for functional domains). In order to reduce the number of features, feature selection was performed. Finally, 234 features were selected. The importance of these features was ranked by F-score. Among the top 10 features, 4 were amino acids, namely cysteine, threonine, glycine, and isoleucine. Their ranks were 1, 2, 3 and 9, indicating that amino acid composition was the most discriminative features in our model. The other six were functional domians, including IPR011009 (protein kinase-like domain), IPR024736 (oestrogen-type nuclear receptor final *C*-terminal domain), IPR011011 (zinc finger FYVE/PHD-type), IPR001452 (src homology-3 domain), IPR018359 (bromodomain) and IPR004367 (cyclin *C*-terminal domain). According to cross validation, the performance of SVM model was slightly improved by feature selection, from 90.5% to 91.9%. To further improve the predictive accuracy of our model, we also employed an upsampling procedure. This procedure results in a more accurate representation of the negative set. As expected, when an upsampling factor of 4 or 5 was used, the accuracy was as high as 98%.

PR is a member of the nuclear receptor superfamily of ligand-activated transcription factors [19]. Ligand-occupied PR is recruited to DNA to activate or repress transcription [20]. Transcriptional specificity of PR depends on the availability of PPI cofactors in target cells [21]. Under gene ontology, these PPI cofactors belong to the category of transcription regulatory activity. Known PR PPI cofactors include EP300 [22], NCOA1-3 [23,24], and NCOR1-2 [25]. PR can also interact with other transcription factors with nucleic acid binding activity, such as SP1 [26], AP1 [27], FOXO1 [28] and STAT3 [29], to modulate their transcriptional activity. In addition, it has been shown that the activity of PR can be modulated by phosphorylation at Ser345 [26]. Therefore, protein kinases present another type of PR-interacting proteins. Within gene ontology, proteins with transcription regulatory activity, nucleic acid binding activity or kinase activity may be cross-classified into the category of signal transduction activity. By applying our SVM model, we scanned the whole genome to find new human proteins that potentially interact with PR. We obtained a total of 827 new candidates. In general, the majority of new candidates fell into the four categories as mentioned above. Examples included the following: TRRAP, TRIM28, TRIM66, CREBBP and NSD1 in the category of transcription regulatory activity; PPARG and NFKB1-2 in the category of nucleic acid binding activity; and EGFR, AKT1-3 and JAK1-3 in the kinase activity category. Interestingly, we discovered a novel category termed cytoskeletal activity which was unique to the newly predicted PR-interacting proteins. Free PR is located in the cytoplasm. Upon progesterone binding, ligand-occupied PR is transported into the nucleus [20]. Considering that cytoskeletal proteins play an important role in transportation, we suspected that the interaction between PR and cytoskeletal proteins may facilitate PR translocation from cytoplasm into nucleus. Our analysis might provide new clues for PR regulation and function, thus deserving further investigation.

In conclusion, our work demonstrated that it is possible to reliably prediction of PPI partners of a particular protein by using the SVM model. When applying to PR, we achieved an accuracy of 91.9%, sensitivity of 92.8% and specificity of 91.2%. Notably, our method requires simple input data and could be used in a wide variety of applications. We believe that our method will be useful in guiding biomedical experiments.

## 4. Materials and Methods

### 4.1. Data Collection and Data Set Construction

Known progesterone receptor (PR, UniProt: P06401)-interacting proteins were collected from PINA2 database [30], which integrates up-to-date protein-protein interactions available in IntAct [31], BioGRID [32], MINT [33], DIP [34], HPRD [35] and MIPS [36]. A total of 71 interacting proteins were retrieved. Considering that the predictive model will possibly be biased to homologous sequences in the training set, we removed homologous sequences with more than 70% identity by using CD-HIT [37]. Since the non-interacting proteins were not readily available, we used a random way to select negative proteins [38]. Two requirements were met: (i) the non-interacting proteins cannot appear in the positive data set, or exhibit more than 70% identity with any proteins in the positive data set by CD-HIT-2D [37]; and (ii) the number of negative proteins is equal to that of positive proteins. We repeated the negative data set construction procedure 100 times. The influence of random selection was evaluated. Additionally, positive data set was upsampled by a factor of 1 to 5. We then constructed larger negative data sets by random selection. The robustness of upsampling factor was evaluated.

For independent testing, we collected another two data sets for androgen receptor (AR UniProt: P10275) and estrogen receptor (ER, UniProt: P03372). The same criteria were employed as described above. There were 299 interacting protein and 296 non-interacting proteins in final testing data set for AR. The final testing data set for ER contained 547 interacting protein and 531 non-interacting proteins.

### 4.2. Feature Extraction

For amino acid composition, each protein sequence was represented using a vector {*x_i_*, *i* = 1, 2, …, *n*}. The vector *x_i_* has 20 elements corresponding to the occurrences of 20 amino acids normalized with the total number of residues in the protein. We also considered amino acid dipeptide composition with 400 elements specified the occurrences of 400 amino acid dipeptides normalized with the total number of dipeptides in the protein. We finally excluded dipeptide composition in the feature vector, because it did not improve the performance of the SVM classifier.

The interacting domain between proteins is indicative of PPI. Previous studies have shown the feasibility of using functional domain information to predict PPI [39,40]. In this work, protein domains were investigated as features for classifying PR-interacting proteins from non-interacting ones. We retrieved domain information of each protein in the training data set by referring to its corresponding InterPro records [41] in the UniProt database [42]. For each protein, a feature vector of ones and zeros was constructed: one for presence and zero for absence of a certain functional domain. The feature selection tool for libsvm was used to reduce the number of features and the relative importance of each feature was calculated based on F-score (http://www.csie.ntu.edu.tw/~cjlin/libsvmtools/fselect/fselect.py).

### 4.3. Model Construction

The software libsvm 3.20 (http://www.csie.ntu.edu.tw/cjlin/libsvm/) was employed in this work. The radial basis function (RBF) was chosen as the kernel function, which is defined as:

(4) k(u,v)=exp(−γ||u−v||)

where *u* and *v* are two data vectors and γ is the kernel width parameter. We evaluated the predictive performance of the constructed model by 5-fold cross validation. During this process, the training data set was divided into five equal or nearly equal groups. In one round of cross validation, one subgroup was regarded as the test set, and the remaining four subgroups were treated as the training set. The cross-validation process was repeated five rounds, with each of the five subgroups used as the test set in turn. Then, all the results were combined to produce a single estimation. During the process of 5-fold cross validation, the regularization parameter *C* and the kernel width parameter γ were optimized to maximize predictive accuracy using a grid search approach. Finally, the parameters that yielded the highest accuracy were employed to construct the predictive model. ROC curve and AUC were calculated during 5-fold cross validation using a MATLAB script (http://www.csie.ntu.edu.tw/~cjlin/libsvmtools/roc/plotroc.m).

### 4.4. Functional Clustering Analysis of PR-Interacting Proteins

For functional clustering analysis, we adopted the gene ontology terms defined by MGI GOslim (http://www.informatics.jax.org/gotools/MGI_GO_Slim.html). The ontology covers three categories: biological process, cellular component and molecular function. Known PR-interacting proteins and newly predicted PR-interacting proteins were mapped to GOslim terms under the molecular function category.

## Supplementary Materials

Supplementary materials can be found at http://www.mdpi.com/1422-0067/16/03/4774/s1.
